# Traditional healers as client advocates in the HIV-endemic region of Maputo, Mozambique: results from a qualitative study

**DOI:** 10.1080/17290376.2021.1909492

**Published:** 2021-04-26

**Authors:** Radhika Sundararajan, Patricio V. Langa, Trisha Morshed, Sandra Manuel

**Affiliations:** a Emergency Medicine, University of California, San Diego, CA, USA; b Faculty of Arts and Social Sciences, Universidade Eduardo Mondlane, Maputo City, Mozambique; c Emergency Medicine, Banner Medical Center, Vituity Physician Group, Sun City, AZ, USA; d Faculty of Education, Universidade Eduardo Mondlane, Maputo City, Mozambique

**Keywords:** Traditional healers, Mozambique, social capital, qualitative

## Abstract

Traditional healers are commonly utilised throughout sub-Saharan Africa instead of – and in concert with – biomedical facilities. Traditional healers are trusted providers and prominent community members and could be important partners in improving engagement with HIV services in endemic contexts. Our study sought to understand the roles of healers in the urban setting of Maputo, Mozambique, where HIV prevalence is high and testing rates are low. Qualitative data were gathered through minimally structured interviews with 36 healers. Analysis followed an inductive, grounded theory approach. Data reveal three themes relevant to improving engagement with HIV services in this endemic region: (1) healers have positive attitudes towards biomedicine; (2) healers advocate for their sick clients and (3) clients are reticent to present to biomedical facilities. Healers describe their roles as ‘cooperative’ with biomedical providers to provide healthcare for their clients. Results suggest that healers could be considered *critical enablers* to effective HIV programmes in communities. They have social and symbolic capital that positions them to beneficially influence clients and are natural partners for interventions to improve uptake of HIV services.

## Introduction

Throughout sub-Saharan Africa, traditional healers play a central role in communities; the WHO estimates that 80% of the general population of sub-Saharan Africa utilises these practitioners (World Health Organization, 2002). In Mozambique specifically, they play an even more crucial role in managing health complaints. With independence in 1975 came a massive exodus of Mozambique’s physicians, many of whom were under contract with the Portuguese colonial government. Much of the country’s health infrastructure was additionally decimated by a subsequent 16 year-long civil war. Now, Mozambique faces a dearth of physicians, with only 5 for every 100,000 people (UNAIDS, 2018), among the lowest densities of physicians in the world. Healthcare facilities and healthcare workers are also sparsely distributed around the country, with less than 50% of the population having regular access to health services (Global Health Workers Alliance, 2013); people must travel long distances to access healthcare at biomedical facilities. There are no official statistics describing the number of traditional healers in the country, but they are unofficially estimated at >80,000 (Green, 1999). The study site of Maputo is the nation’s capital, a city of ∼1.7 million people, home to the nation’s largest hospital (Maputo Central Hospital) and the region of Mozambique with the greatest coverage of healthcare facilities (Anjos Luis dos & Cabral, 2016). However, Maputo is home to a large number of healers, demonstrating that their importance is not limited to contexts with a low density of biomedical facilities.

Ethnographic and community-based research from sub-Saharan Africa suggests that healers may be favoured over biomedical practitioners because they are considered custodians of indigenous knowledge, and commonly hold positions of authority in their communities (Homsy, King, Balaba, & Kabatesi, 2004; James, Wardle, Steel, & Adams, 2018). As such, their evaluations and recommendations could have cultural appropriateness that biomedicine may not, considering factors outside of biomedical physiology that may contribute to symptoms, such as ancestral unrest, family conflicts or social transgressions (Green, 1999; Green, Zokwe, & Dupree, 1995). Prior research has also demonstrated that healers may be perceived as more trustworthy than biomedical providers (Hampshire, Hamill, Mariwah, Mwanga, & Amoako-Sakyi, 2017; Homsy et al., 2004; King & Homsy, 1997; Moshabela et al., 2017; Taylor, Dolezal, Tross, & Holmes, 2008) and favoured as a result of prior negative experiences at healthcare facilities (Morris, 2001; Sundararajan, Kalkonde, Gokhale, Greenough, & Bang, 2013), including ‘stock outs’ of medications, malfunctioning machines, long wait times to access service and perceived breaches of confidentiality by biomedical providers.

The utilisation of traditional healers is particularly relevant to the human immunodeficiency virus (HIV) epidemic in Mozambique. Mozambique is among the 10 countries with the highest HIV burden. The most recent national survey estimates HIV prevalence among sexually active adults at 12.6%, with 2.2 million people living with HIV/acquired immunodeficiency syndrome (AIDS) (UNAIDS, 2018). In Maputo, prevalence is much higher (16.9%) and is where 9% of all Mozambicans living with HIV reside (Mozambique Ministry of Health, 2018; PEPFAR, 2016). Despite this, lifetime HIV testing rates in Mozambique are very low (37% for women and 19% for men) (Mozambique Ministry of Health, 2018). The country’s long civil war has also impacted the current population-level skew that is particularly relevant to the HIV epidemic: 51% of the current population is of sexually active age (Global Health Workers Alliance, 2013). Studies throughout sub-Saharan African have demonstrated that traditional medicine is frequently utilised by HIV-infected patients, both prior to and following diagnosis (Hughes et al., 2012; Moshabela et al., 2017; Moshabela, Pronyk, Williams, Schneider, & Lurie, 2011; Wanyama et al., 2017). Prior research in Mozambique has demonstrated that healer utilisation is associated with delays in HIV diagnosis (Audet et al., 2014), though there are no data for urban centres where biomedicine is readily accessible.

Mozambican healers work as spiritualists (*Nhamussoro*), herbalists (*Nhangarume*) or birth attendants. Spiritual healers claim the ability to identify and remove curses or address ancestral unrest through rituals or incantations. In addition to this specialisation, they also utilise herbs to heal (via ingestion, ‘bath’ or ‘vaccination’, where herbs are rubbed into a small incision made in the skin). While spiritual healers use herbs in their treatments, herbalists deal exclusively with herbal remedies. Birth attendants provide prenatal and obstetrical care to women and employ ‘traditional’ methods for birthing outside of biomedical facilities. Traditional healer services are formally endorsed by the Ministry of Health and are largely overseen by the Association of Traditional Healers [Associação dos Médicos Tradicionais de Moçambique, AMETRAMO]. AMETRAMO-affiliated practitioners have standardised treatments and fees for service, and attend periodic training sessions, in cooperation with the Ministry. Membership in AMETRAMO is not compulsory; many traditional healers are not members of this group, and therefore are not held to the same training and practice regulations.

Little is known about traditional healers who practice in urban settings where biomedical resources are more easily accessible for the general population, and whether delivering healthcare within a context of relatively high resources impacts their engagement with biomedicine or knowledge about HIV services. This qualitative study addresses this gap in knowledge by (1) considering content and sources of HIV-related knowledge among traditional healers practicing within Maputo City; (2) characterising healer attitudes towards biomedicine and (3) describing actions taken by healers when caring for a client they suspect to be HIV-infected.

## Materials and methods

### Study design

**Figure 1.**
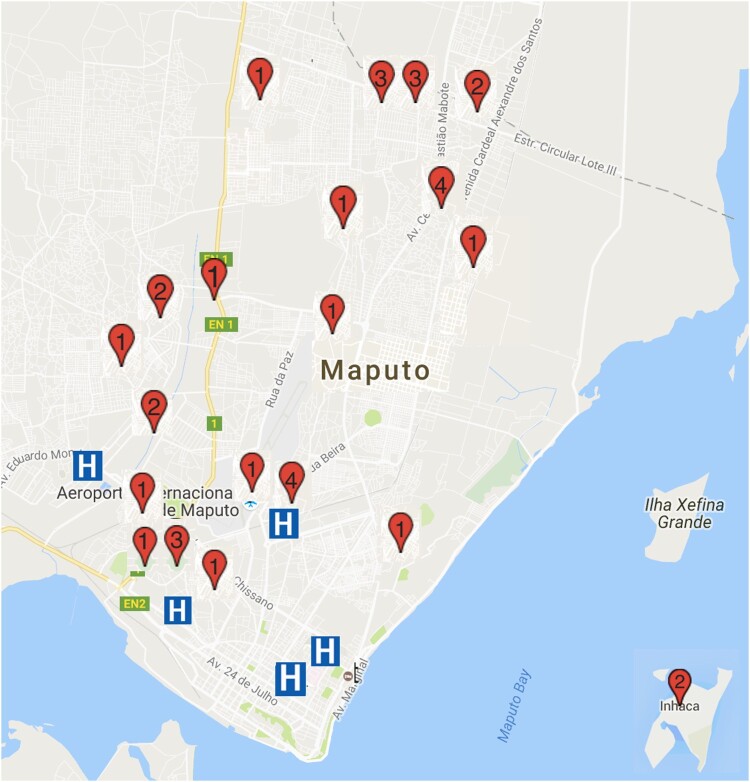
Map of participant practice locations around Maputo City. Note: Red pin locations and numbers indicate location and number of participants from this neighbourhood. Blue ‘H’ demonstrates location of hospital. Map Data: © 2016 Google. The street map and hospital locations are freely available on maps.google.com. Permission was not required to reproduce this map image.

We enrolled 36 traditional healers practicing throughout Maputo City District between April and November 2016 (Figure 1). A qualitative study design was employed, with semi-structured interviews to elucidate current practices and beliefs about HIV, and interfaces with biomedicine. An interview guide was created in English, translated into Portuguese and the local language (*Changana*) and back-translated into English to verify the preservation of meanings. All participants provided socio-demographic information, including practice specialty, years of practice, the highest level of education, client volume and income. The interview guide was piloted with two traditional healers in March 2016, whose responses are not included in this analysis.

### Participant recruitment and sampling

During recruitment, research assistants introduced themselves and the study as affiliated with the local university; care was taken not to represent the study or its staff as representatives of hospitals or clinics. Approximately half of our participants (*N* = 15) were recruited in cooperation with AMETRAMO. The remaining participants (*N* = 21) were recruited with assistance from healers and community members, using a snowball sampling approach. Participating healers and patients seeking care from participating traditional healers were asked to provide contact information for traditional healers they knew of in Maputo City. This recruitment strategy sought to maximise the representation of healers throughout the study region and avoid including only healers who may provide responses that solely reflect training and practices consistent with the AMETRAMO organisation.

This purposive sampling strategy aimed to include ‘information-rich’ cases to maximise the quality of our qualitative data by including participants with a broad range of knowledge and experience with our topics of interest (Palinkas et al., 2015). A sample size of *N* = 36 was guided by the concept of qualitative *data saturation* where interviews no longer reveal substantially new or relevant content, and categories are sufficiently developed based on completed interviews (Corbin & Strauss, 2014). Transcripts were analysed throughout the data collection period; iterative engagement with the dataset allowed timely communication among the research team to direct interviews, ensuring that emerging themes could be explored and well developed.

### Data collection

Healers were eligible for participation if they were (1) 18 years of age or older; (2) recognised by the community as a practitioner of traditional medicine and (3) able to provide informed consent in Portuguese or Changana. Semi-structured interviews lasted approximately 1 hour and were conducted by four Mozambican research assistants (two females and two males) with educational backgrounds in behavioural science and fluent in English, Portuguese and Changana. Research assistants used an interview guide to ensure that the following core topics were discussed consistently: (1) HIV knowledge (e.g. HIV symptoms, modes of transmission, risk factors and diagnosis/treatment/cure) and sources of knowledge; (2) prior experiences and attitudes towards biomedicine (e.g. biomedical resource utilisation with prior illnesses, trustworthiness of biomedical providers and current thresholds for referring clients for biomedical care) and (3) current traditional treatments provided to clients (e.g. approaches to healing, which ailments/illnesses can be treated with traditional medicine). Research assistants were also encouraged to explore novel or interesting topics that arose in the course of the interview.

### Data management and analysis

Interviews were transcribed by the interviewer into Portuguese (translated if the interview was conducted in Changana) and then translated into English. One author (PVL) ensured the validity of Changana, Portuguese and English translations through random spot-checking, as well as a targeted review of transcripts (when the English translation was unclear or confusing, for example), against interview audio. Two authors (RS and TM) reviewed all English transcripts for content relevant to HIV-related knowledge and practices among traditional healers, within 1 week of interview completion. The first author analysed interview data using an interpretive phenomenological approach to data analysis (Smith, Flowers, & Larkin, 2009; Smith & Osborn, 2015), as the goal of this study was to explore participants’ own experiences and perspectives on traditional medicine, HIV-services and biomedical care. Through open coding and constant comparative analysis (Glaser & Strauss, 1967), preliminary categories were formed. These categories were reviewed, sharpened, refined through data triangulation and grouped with the generation of a final set of codes. At the completion of data collection, all transcripts were re-reviewed by applying the final set of codes to identify major themes illustrating HIV-related knowledge and practices among healers in Maputo, Mozambique. Using an axial coding approach, we identified connections and relationships between codes and then employed selective coding to refine the relationships between concepts and identify overarching themes. Example(s) from our dataset are presented in the ‘Results’ section that best illustrates these themes. NVIVO 10.0 (QSR International) was used for qualitative data management, but not in the creation of codes. Demographic characteristics of participants are summarised with basic descriptive statistics.

### Ethical considerations

This research was approved by the Institutional Review Board of the University of California, San Diego (Protocol #150062, PI: Sundararajan) and the Biomedical Ethics Committee of the Universidade Eduardo Mondlane Faculty of Medicine. All participants were volunteers and provided with informed consent, both written and reviewed verbally to ensure comprehension among those participants with limited literacy. Healers were remunerated with 1000 meticais (∼$15 USD) as compensation for time lost to revenue generation. This remuneration rate was set by AMETRAMO and is the standard rate for members participating in research studies. The same remuneration was extended to non-AMETRAMO participants in the interest of equity. All audio and data files were stored in password-protected electronic files, accessible only to study staff.

## Results

### Characteristics of participants

Table 1 shows characteristics of our participants. In our sample, the vast majority self-identified as spiritual healers (*N* = 34, 94%), and two identified as pure herbalists. There were no birth attendants in our sample, potentially due to the proximity of biomedical facilities for prenatal/obstetrical services. All 36 healers approached for enrolment agreed to participate in the study. Consistent with other research involving healers in Mozambique and South Africa, our participants are largely female (67%).

**Table 1.** Characteristics of study participants (*N* = 36)

Participant Characteristic	Value	Standard Deviation
Age (in years)	48 (mean)	12.2
Gender	67% Female (*N* = 24)	n/a
AMETRAMO members	42% (*N* = 15)	n/a
Highest level of education (grade)	Grade 4.5 (median)	9.0 (IQR)
Weekly income (in USD)	$33.41 (mean)	$17.41
Weekly client volume (in persons)	4.6 (mean)	2.2
KQ-18 HIV knowledge score	11.2 (mean; 18 maximum score)	3.4

### Interview themes

We note three major themes within our interviews that are relevant to understanding the roles of traditional healers in this high HIV-endemic context: (1) praise for biomedicine; (2) advocacy for sick clients and (3) client reticence for biomedical testing.

#### Praise for biomedicine

Among our sample, healers report uniformly high praise for biomedicine and biomedical providers. Participants describe biomedicine as ‘curing’ and ‘as important’ as traditional medicine in its healing properties. All participants report the use of hospitals and clinics when they and their families fall ill. Specifically, participants admire biomedicine for its ability to ‘look within’ for ‘internal’ maladies through the use of serum testing and radiographs, while traditional approaches cannot. Some describe biomedicine as ‘complementary’ to traditional healing, and themselves ‘collaborators’ with biomedical providers. One participant describes a common process of clients tacking between the two realms:

For the natural ones [biomedical illnesses], I tell the patient to go to hospital. But it is important to say that there are some cases in which the patient had been to the hospital and the disease was not diagnosed. So when they come to me, I remove the evil spirit that was preventing the diagnosis of the disease, and then I send back the patient to the hospital. I also encourage the patient by saying that s/he will get well soon and what s/he needs to do is to follow the hospital doctor’s instructions. (Participant 305, Male, 53 years old)

The healer in this case describes a context of *medical pluralism*, where both traditional and biomedical forms of healing are utilised by the client. He is encouraging the client to adhere to instructions provided by biomedical providers, suggesting that healers do not conceive of hospitals or clinics as competitive entities, nor are they distrustful of their recommendations. Rather, they offer support and encourage the client’s biomedical utilisation.

However, some participating healers reported that they felt ‘scorned’ by biomedical providers, who did not reciprocate the collaborative attitudes the healers espouse. Some participants believed that physicians did not respect the work that healers perform for their patients and communities, which should also be considered a divine ‘gift’. One participant states:

What I would like to see happen in our country, between conventional and traditional medicine, is respect and understanding of each other. We traditional healers are looked down on by medical doctors. They say they went to a University, forgetting that I also have my gift given by God. (Participant 205, Male, 36 years old)

Healers perceive that biomedical providers hold negative opinions of their work, and they are reticent to disclose their vocation if they accompany clients to receive care at biomedical facilities: ‘We face problems because in hospitals we are still looked down on. If they know you are a traditional healer, there’s so much scorn. Not many will welcome you. They forget that we have knowledge like they do’ (Participant 203, Female, 59 years old).

#### Advocating for their clients

Participants describe behaviours that facilitate client utilisation of biomedicine, particularly for those presenting with symptoms of AIDS. They report familiarity of symptoms of HIV infection as those related to AIDS (weight loss, chronic cough, alopecia, diarrhoea, chronic skin rashes/wounds); these clients are immediately referred to the hospital. Our results support the concept that healers are *advocates* for their clients and consider themselves to be working in cooperation with biomedical providers. Advocacy behaviours include escorting patients to the hospital themselves, phoning in ‘referrals’ to hospitals, ‘begging’ or otherwise persuading clients to visit the hospital. Such advocacy is underlain by feelings of ‘love’ and ‘obligation’, and acting in the best interests of clients, whom they consider ‘family members’. A healer describes her relationship with one of her clients, who was recently diagnosed as living with HIV:

It still secret [that my client is living with HIV]. No one else knows apart from me, even his relatives. So now I have become his relative, because I am the one who escorts him to the clinics, a thing which his relatives do not do … When we were at the hospital, the only thing that the patient was saying is that he ‘preferred to die’. Then I asked, ‘how about the two daughters that you have?’ I told him that he is still young and he can live a long life if only he takes care of himself and starts to medicate. It took a lot of effort in order to enroll [in HIV care] at the hospital for him to start medications, and sure enough, I managed to help him do it. He is medicating and now he shows some good signs. (Participant 301, Female, 49 years old)

This participant elaborates on a sense of responsibility in convincing a sick-appearing client to go to the hospital:

He did not welcome the news [of going to the hospital], but since we fight for people’s lives, we need to be bold enough to do so. We cannot mess up with these diseases, especially AIDS … Diseases like this one, they need medical attention. (Participant 208, Female, 45 years old)

Another healer describes his role in the lives of his clients: ‘The job we have is so important, I say. Because from the traditional perspective of healing there is a lot that we are able to help a lot of people with … We are part of what helps a human being’ (Participant 202, Male, 59 years old).

#### Clients ‘run’ from biomedicine

Traditional healers in our sample report that their clients frequently object to their referrals for biomedical evaluation and testing and will ‘run away’ from the healer to avoid this outcome. Healers ‘know by looking at someone’ if the client is ‘sick or not’; sick clients warrant urgent referral to hospitals. However, they have learned to approach these clients ‘carefully’ to avoid causing them to flee:

We have learned now. We know how to talk to them, because a person in those conditions [when clients are sick] is so sensitive. If we do not attend to him here, he will not even get to a hospital … I will handle [that client] carefully, because you cannot communicate aggressively to the patient. If you do so, he will run away … so, you be tender as you inform him, until he goes to hospital. (Participant 203, Female, 50 years old)

Our participants attribute client resistance to fear of biomedical testing (for HIV, specifically). One participant reports, ‘Many times, people say that when a traditional healer fails to help you, it means you are dead already’ (Participant 202, Male, 59 years old). This saying underlies the concept that traditional practitioners are trusted and preferred for their capacity to *heal*, while visits to biomedical facilities may be associated with bad outcomes and death. Another healer suggests that their clients prefer traditional medicine as a means of avoiding their medical diagnoses: ‘When they come here, sometimes they are avoiding going to hospitals, or they have been there and were diagnosed with HIV and he hides it and come to us, and you ask him back to hospital – again he runs away’ (Participant 204, Female, 66 years old). Healers also suggest that clients avoid biomedical facilities due to perceived poor quality of services, such as ‘long queues’ or ‘defective machines’, and the fact that practitioners are ‘not friendly to patients in our hospital. I think when one thinks of having his HIV status know by those professionals, I thinks it scares him’ (Participant 213, Female, 35 years old).

## Discussion

This qualitative study elucidates experiences of healers practicing in Maputo, Mozambique, an HIV-endemic region, which is also home to the highest density of biomedical resources in the country (Anjos Luis dos & Cabral, 2016). Our results demonstrate that Maputo is a medically pluralistic context with cyclical/concurrent traditional and biomedical use within one’s therapeutic itinerary. Participants state that clients ‘run’ from biomedical facilities, potentially due to mistrust of services and providers, and preference for culturally competent traditional healing. Despite low opinions and avoidance by clients, our healer participants report high regard for biomedicine. They consider themselves as collaborators with biomedical providers and advocate for clients to escalate care, often facing resistance from clients whom they refer to the hospital. This provides a unique, holistic arrangement, where biomedical and traditional approaches to healing are accepted and encouraged.

Our research demonstrates that traditional healers function in their communities as informal client advocates. Their advocacy actions are based on their sense of obligation to clients, whom they consider to be close, like family members. Healers develop strategies to overcome client resistance to HIV testing, by approaching them in a ‘tender’ manner, through developing rapport and providing reassurance. They ‘refer’ and accompany clients to formal healthcare facilities to support HIV service engagement. The behaviours and strategies described by our participants mirror ‘treatment advocate’ programmes implemented by biomedical facilitates and AIDS service organisations (Mutchler et al., 2011), which have been shown to improve medication adherence and self-efficacy for people living with HIV (Bogart et al., 2012).

Furthermore, our results describe an important factor contributing to low rates of HIV testing in Mozambique: traditional healer client reticence for biomedical testing. This describes a bottleneck in the cascade of HIV care. If HIV-infected patients are not identified through voluntary testing, they cannot be enrolled into care. This is particularly problematic because early diagnosis and initiation of antiretroviral therapy is crucial to preventing transmission and optimising health outcomes (The INSIGHT START Study Group, 2015). Our results also speak to the phenomenon of the widening gender gap in life expectancy among HIV-infected populations in sub-Saharan Africa, where men have two-fold higher mortality rates than women (Bor et al., 2015). These ‘missing men’ (Tsai & Siedner, 2015) may be clients who ‘run away’ from hospitals and biomedical facilities. Efforts to improve engagement with male populations in HIV-endemic regions should therefore consider those who visit healers, and how collaborative efforts may overcome reticence towards engagement with HIV services. This holistic approach to client health explains why healers maintain positions of authority in their communities. This is no less important in the urban, capital city than it is for clients living in a rural district with no local hospital.

Based on our results, we suggest that traditional healers could be considered one of the ‘critical enablers’ (UNAIDS, 2012) of effective responses to the AIDS epidemic in Mozambique, if not throughout much of sub-Saharan Africa where they practice. Critical enablers are stakeholders who can ‘overcome major barriers to [HIV] service uptake, including social exclusion, marginalization, criminalization, stigma and inequity. Critical enablers are crucial to the success of HIV programs in all epidemic contexts’ (UNAIDS, 2012). Our results, and others, indicate that healers are preferred providers due to positions of respect and trust in their communities (Green, 1992), their ability to provide holistic and culturally appropriate care (O’Brien & Broom, 2014; Taylor et al., 2008), and are relied upon in regions that lack available biomedical resources (Morris, 2001).

Traditional healers possess *social capital*, amassed through their engagement in ‘networks of social acquaintance and recognition’ (Bourdieu, 1986). These networks of interactions and prestige also generate *symbolic capital*, where ‘power is granted to those who have obtained sufficient recognition to be in a position to impose recognition’ (Bourdieu, 1989). Our data demonstrate how healers mobilise their social and symbolic capital to benefit clients through their advocacy – facilitating access to healthcare resources and encouraging clients to undergo HIV testing – through a cooperative approach to healing. As such, healers are gatekeepers to communities with poor biomedical engagement. National and international responses to ending the HIV epidemic by 2030 (UNAIDS, 2014) must consider the impact of these practitioners within their communities. Our data, like others from varying contexts (Audet, Hamilton, Hughart, & Salato, 2015; Homsy et al., 2004; King & Homsy, 1997), demonstrate that healers have overwhelmingly positive attitudes towards biomedicine and are eager to engage in cooperative approaches to patient care. For example, Moshabela et al. (2016) found that traditional healers boosted the impact and acceptability of an HIV test-and-treat intervention through educating clients on HIV-related stigma and supporting linkage to HIV care. There have been a few successful partnerships with traditional healers to expand access to health screening programmes in medically pluralistic contexts. These initiatives have included trainings for healers to deliver counselling and facility referral for HIV (Homsy & King, 1996; King & Homsy, 1997), tuberculosis (Peltzer, Mngqundaniso, & Petros, 2006) or malaria testing (Makundi, Malebo, Mhame, Kitua, & Warsame, 2006), provide mental healthcare (Shoesmith et al., 2020; Solera-Deuchar, Mussa, Ali, Haji, & McGovern, 2020) or to increase uptake of prenatal care (Audet et al., 2015).

Healers are willing to work in collaboration with biomedical providers, researchers and public health officials and have the social capital to benefit clients by advocating for them to receive biomedical care. However, healers perceive disrespect from biomedical providers, who do not recognise traditional healers as skilled healthcare providers, and do not reciprocate the cooperative spirit described by our study participants. Negative attitudes towards traditional medicine have been described as the primary barrier to true collaboration between traditional and biomedicine, as biomedical providers repeatedly downplay the skills and contributions of traditional healers (Akol, Moland, Babirye, & Engebretsen, 2018; Audet et al., 2013; Gall, Anderson, Adams, Matthews, & Garvey, 2019). Studies have described how biomedical providers express distrust and disapproval of traditional medicine in interactions with their patients (Audet et al., 2013; Gall et al., 2019; Puoane, Hughes, Uwimana, Johnson, & Folk, 2012). Further work to develop formal, functional partnerships between traditional and biomedical providers must consider the bias of those working in formal healthcare settings and develop strategies to overcome unilateral fear and mistrust.

### Limitations

We recognise some limitations of our study. Qualitative data are not intended to be largely generalisable; rather, they provide detailed and highly contextual information about a population of interest. As such, we acknowledge that our qualitative findings may be specific to a population of traditional healers practicing in a relatively well-serviced city (Maputo, Mozambique). We do, however, note some similarities with studies of healers practicing in more remote areas of sub-Saharan Africa, particularly insofar as we describe positive attitudes towards biomedicine and willingness to collaborate with biomedical providers. Our data reflect perspectives and experiences of traditional healers, and more work is needed to assess how their actions impact experiences of biomedical providers and healthcare outcomes among clients with pluralistic itineraries.

## Conclusions

Our data include findings that can inform efforts to meet the Joint United Nations Programme on HIV/AIDS 95-95-95 benchmarks to end the epidemic (Joint United Nations Program on HIV/AIDS (UNAIDS, 2015)). We present three important themes relevant to the roles of traditional healers in HIV-endemic regions: (1) positive attitudes towards biomedicine that underlies cooperative healing for their clients; (2) advocacy for clients and (3) client reticence for biomedical testing. Healers practicing in these regions could be considered *critical enablers* to effective HIV programmes. Their social and symbolic capital positions them to beneficially influence clients. They are natural partners to improve uptake of HIV services in communities with poor biomedical engagement.
